# Teaching and learning strategies in Home Enteral Nutritional Therapy: Knowledge gains perceived by caregivers

**DOI:** 10.1590/1518-8345.6032.3888

**Published:** 2023-03-27

**Authors:** Maria Gabriela Afonso, Luiz Henrique Arroyo, Amanda Adabo Gastaldi, Ana Carolina Belmonte Assalin, Mellina Yamamura, Fernanda Berchelli Girão

**Affiliations:** 1 Universidade Federal de São Carlos, Departamento de Enfermagem, São Carlos, SP, Brazil.; 2 Scholarship holder at the Fundação de Amparo à Pesquisa do Estado de São Paulo (FAPESP), Brazil.; 3 Universidade de São Paulo, Escola de Enfermagem de Ribeirão Preto, PAHO/WHO Collaborating Centre for Nursing Research Development, Ribeirão Preto, SP, Brazil.

**Keywords:** Health Education, Enteral Nutrition, Caregivers, Home Care Services, Learning, Teaching, Educação em Saúde, Nutrição Enteral, Cuidadores, Cuidado Domiciliar, Aprendizagem, Ensino, Educación en Salud, Nutrición Enteral, Cuidadores, Cuidado Domiciliario, Aprendizaje, Enseñanza

## Abstract

**Objective:**

to evaluate how different educational strategies contribute to knowledge gains perceived by caregivers of people using Enteral Nutritional Therapy.

**Method:**

a quasi-experimental study conducted in two stages: the first one included an interactive lecture class (LC) and the second was carried out in two groups: *in-situ* simulated skills training (ST) and reading of an educational booklet (EB). The caregivers answered a self-administered questionnaire to assess knowledge before and after the interventions; for the analysis, a generalized linear model with Poisson distribution was proposed and the comparisons were carried out using orthogonal contrasts.

**Results:**

the participants were 30 caregivers; evidence of a difference in knowledge between the t_1_and t_0_ moments is evidenced. The analysis of the final comparison about the knowledge gain between the EB and ST groups, according to Student’s t, evidenced an estimated difference of -1,33, with 95% CI (-4.98; 2.31) and p-value=0.46.

**Conclusion:**

knowledge was further increased between the t_1_ and t_0_ moments, when compared to the t_2_ and t_1_ moments in both groups. When compared, we cannot conclude that one of the groups changed more than the other in relation to moment t_0_ and t_2_; thus, the study evidenced the knowledge gain after all the educational strategies in both groups.

## Resumo

**Objetivo:**

avaliar como diferentes estratégias educativas contribuem para ganhos de conhecimento percebidos por cuidadores de pessoas em uso da Terapia Nutricional Enteral.

**Método:**

estudo quase-experimental realizado em duas etapas; a primeira contemplou uma aula expositiva dialogada (AE) e a segunda aconteceu em dois grupos: treino de habilidades (TH) simulado *in situ* e leitura da cartilha educativa (CE). Os cuidadores responderam um questionário autoaplicável para avaliação de conhecimentos em pré e pós-intervenções; para a análise foi proposto um modelo linear generalizado com distribuição Poisson e as comparações foram realizadas por contrastes ortogonais.

**Resultados:**

participaram 30 cuidadores, observou-se evidência de diferença de conhecimento entre os tempos t_1_ e t_0_. A análise da comparação final sobre o aumento do conhecimento entre os grupos CE e TH, por teste t-Student, evidenciou uma diferença estimada de -1,33, com IC 95% (-4,98; 2,31) e valor de p de 0,46.

**Conclusão:**

ocorreu uma maior elevação de conhecimento entre os tempos t_1_ e t_0_, quando comparada os tempos t_2_ e t_1_ em ambos os grupos. Quando comparados, não podemos concluir que um dos grupos mudou mais que o outro em relação aos tempos t_0_ e t_2_; assim, o estudo evidenciou o ganho de conhecimento após todas as estratégias educativas nos dois grupos.

## Resumen

**Objetivo:**

evaluar cómo las diferentes estrategias educativas contribuyen a la adquisición de conocimiento percibida por los cuidadores de personas que utilizan Terapia Nutricional Enteral.

**Método:**

estudio cuasiexperimental realizado en dos etapas; la primera incluyó una clase expositiva dialogada (CE) y la segunda se desarrolló en dos grupos: entrenamiento de habilidades (EH) simuladas *in situ* y lectura del folleto educativo (FE). Los cuidadores respondieron un cuestionario autoadministrado para evaluar el conocimiento pre-posintervenciones; para el análisis se propuso un modelo lineal generalizado con distribución de Poisson y las comparaciones se realizaron mediante contrastes ortogonales.

**Resultados:**

participaron 30 cuidadores, había evidencias de la diferencia de conocimiento entre los tiempos t_1_ y t_0_. El análisis de la comparación final sobre el aumento de conocimientos entre los grupos FE y EH, mediante la prueba t de Student, mostró una diferencia estimada de -1,33, con un IC del 95% (-4,98; 2,31) y un valor de p de 0,46.

**Conclusión:**

hubo un mayor aumento del conocimiento entre los tiempos t_1_ y t_0_, que entre los tiempos t_2_ y t_1_ en ambos grupos. Al compararlos, no podemos concluir que uno de los grupos cambió más que el otro entre t_0_ y t_2_; por lo tanto, el estudio demostró que hubo adquisición de conocimiento después de todas las estrategias educativas en ambos grupos.

**Highlights**

(1) Higher proportion of correct answers among the caregivers after the lecture class.

(2) All the caregivers had their knowledge improved at the end of the interventions.

(3) Caregivers with family ties present higher knowledge gains.

(4) Training the caregivers’ abilities with the simulation resulted in better results.

## Introduction

Enteral Nutritional Therapy (ENT) is one of the therapeutic modalities to meet the needs of or recover the patients’ nutritional status, with the possibility of being continued in the home environment^1^.

For the patients that need to use a Nasoenteric Tube (NET) for ENT in the home setting, exposure to the occurrence of adverse events is considerably increased^1^ ^-^ ^2^. Among the most common complications are the mechanical ones, exemplified by displacements, unplanned removal, metabolic obstructions such as gastrointestinal ones, respiratory and infectious diseases such as aspiration pneumonia, contamination and the psychosocial impacts generated to the patient^3^.

During the in-hospital care period, the nurse need to adhere to a holistic and multiprofessional discharge plan, promoting comprehensive care based on technical-scientific, ethical and humanistic knowledge, in order to meet the needs of patients and caregivers^4^. In this sense, home planning should start together with the use of enteral therapy during hospitalization^1^ with educational actions to contribute to the prevention of adverse events and, consequently, to maintenance of the patients’ quality of life^5^.

In view of the above, there is an evident need to develop health education methods to promote greater autonomy in the care of clients using ENT at their homes, training more committed, stable and emotionally prepared caregivers for the resolution and recognition of possible problems, in order to avoid errors and complications resulting from use of this therapy^6^.

A systematic review^7^ reported that caregivers of patients with Home Enteral Nutritional Therapy (HENT). should establish a systematic and personalized process to avoid care failures, requiring careful observation and reflection to avoid complications. The study also showed that the caregivers should communicate with health professionals in a timely manner to ensured safety of flexible strategies; thus, the professionals reinforced regular training of caregivers and ensure adequate knowledge of training^7^. However, there are few published studies on the caregivers’ opinions about what might support them in this home-based care^8^.

Therefore, the objective of this study is to evaluate how different educational strategies contribute to knowledge gains perceived by caregivers of people using Enteral Nutritional Therapy.

## Method

### Study design and locus

A quasi-experimental study^9^ that followed the assumptions set forth in the Revised Standards for Quality Improvement Reporting Excellence (SQUIRE 2.0) guide^10^.

The study was conducted at a University Hospital in a municipality from the inland of the state of São Paulo.

### Participants and recruitment

The caregivers were recruited based on the indications by health professionals from a University Hospital with easy access to the researchers in the city of São Carlos, São Paulo (SP), Brazil. The study population included all caregivers of patients with an indication for HENT at hospital discharge, being invited to voluntarily participate and including those aged 18 years old or over, literate, being caregivers of people over 18 years of age with HENT; able to give their informed consent and ability to understand and communicate in Portuguese, during the data collection period between November 2019 and March 2020.

The sample was comprised by individuals who participated in all the stages of the educational interventions and filled out the sociodemographic data form. 62 (100%) caregivers were invited to participate in the research; of these, 27 (43.5%) refused to take part in reading of the booklet or in the *in situ* simulated skill training and another 5 (8%) withdrew during the process, ending with a final sample of 30 (48%) caregivers.

All the interventions included the same themes. The first one consisted in an interactive lecture class (LC intervention) for the caregivers of patients with indications of HENT. The face-to-face class was developed by the multiprofessional team for nearly two hours, in which issues such as swallowing, dysphagia and anatomy of the digestive system were presented by a speech therapist; food preparation and nutrition guidelines were carried out by the nutritionist; guidelines on patient care on HENT were provided by the nurse and care measure with drug administration by the pharmacist.

Once the aforementioned was finished, the participants were randomly selected to take part in stage two, which was carried out with two groups: group 1, which corresponded to the *in situ* simulated skills training (ST intervention) in a ward room of the medical clinic and group 2, in which reading took place with the support of an educational booklet (EB intervention). Both interventions were based on the interactive lecture class (LC intervention).

For the second stage, the participants were randomly distributed through the open access electronic system on the *Stat Trek* web (http://stattrek.com/Tables/Random.aspx); this process was performed by an independent member of the research team not involved in patient care or data collection.

The caregivers answered an instrument developed in 2019 by the authors based on the frameworks^11^ ^-^ ^16^ on self-perception with gain of knowledge, security, motivation and self-confidence after participating in the educational strategies. The instrument was previously tested with five caregivers, chosen for convenience and not comprising the sample, with the need for small adjustments in order to ease the participants’ understanding.

**Figure 1:**
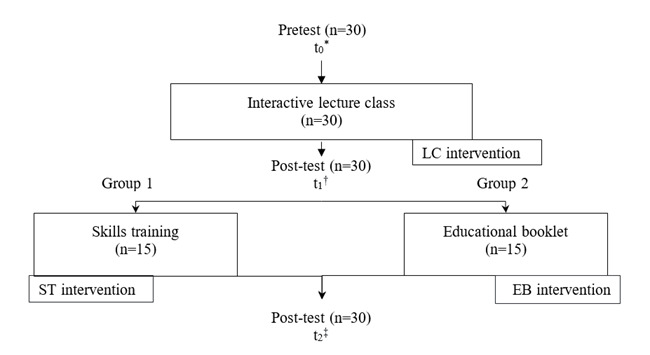
Flowchart corresponding to the stages with distribution of the groups in each intervention. São Carlos, SP, Brazil, 2019 - 2020

Figure 1 presents Flowchart 1, showing the stages and the number of participants in each one. 

*t_0_ = Moment before any intervention; ^†^t_1_ = Moment after the lecture class strategy - LC; ^‡^t_2_ = Moment after other educational strategies (educational booklet - EB and simulated clinical scenario for skills training - ST)

### Data collection tools and methods 

A self-administered questionnaire was used to assess knowledge, consisting of 20 statements about HENT, care measures and possible management of complications, entirely developed by the researchers and applied at moments t_0_, t_1_ and t_2_. There were tree alternatives for each question: one that was true with an assigned score of 1 point; one that was false with a score of 0 and a final “I don’t know” option without any assigned score. At the end of this questionnaire there was an evaluation about the participants’ self-perception in relation to knowledge gain, by means of four alternatives: (1) Very little (safe/motivated/self-confident), (2) Little (safe/motivated self-confident); (3) (Safe/Motivated/Confident) and (4) Very (safe/motivated/self-confident).

After filling in the sociodemographic data form, the self-administered questionnaire on knowledge assessment was applied to all participants at three moments (Figure 1): pre-test (t_0_), before having access to any type of educational strategy; post-test (t_1_), after traditional education and after each of the complementary strategies (t_2_).

### LC intervention: Interactive lecture class (traditional education)

The interactive lecture took place for approximately one hour in a hospital classroom, taught by a multiprofessional team comprised by nurses, nutritionists, speech therapists and pharmacists. A computer for projecting slides was used as a didactic and demonstration resource of all the information about HENT. The presentation addressed the themes of swallowing, digestive system, what is enteral nutrition, access ways, personal and environmental hygiene, ENT preparation (ingredients, supplements, recipe and amounts), administration methods, necessary equipment and utensils, guidelines on patient positioning, as for drip, the most recurrent complications, how to deal with complications (obstruction, displacements, nausea, vomiting, diarrhea, constipation, etc.), guidelines on washing the tube after use for diets, hydration and instructions for taking care when administering medications.

After the interactive lecture class (LC Intervention), the participants were randomly selected in each of both groups (ST Intervention and EB Intervention). 

### ST intervention: Skills training in a simulated clinical setting

The scenario was carried out in a hospital physical environment with a simulated patient using a nasoenteric tube (low-fidelity simulator, male torso type, without any kind of response to the interventions performed) and the following devices: bed, gas network, IV supports, clothes closet (non-sterile swabs, sheets, pillows, pajamas, towels, etc.), headboard, bedside stairs, hand washing sink, measuring tape, enteral nutrition equipment, enteral nutrition bottle and 20 milliliters (ml) and 60 ml syringes.

The simulated skills training aimed at performing activities such as positioning the male torso in elevated decubitus, verification of probe positioning, handling of tools and equipment, adequacy of prescribed drip, administration of enteral nutrition and hydration, fixation, drug administration, dealing with common complications and avoiding probe removal, obstruction and contamination.

### EB intervention: Reading with the support of an educational booklet

The educational intervention was carried out with the booklet on the theme, developed by the authors themselves and validated by judges^17^, which was delivered to each participant. The material consists in 17 items: 1. What is Enteral nutrition?; 2. Materials and equipment; 3. Types of enteral nutrition; 4. Personal and environmental hygiene; 5. Recipe and way to prepare semi-artisan enteral nutrition; 6. List of ingredient substitutions; 7. How to prepare semi-artisan enteral nutrition; 8. Recipe for industrialized enteral nutrition; 9. How to prepare industrialized enteral nutrition; 10. Enteral nutrition administration; 11. Water for hydration; 12. Drug administration; 13. Recommendations; 14. Complications; 15. Enteral nutrition and drug administration via a tube; 16. References and 17. Notes.

Upon delivery, items 2, 10, 11, 12, 13, 14, 15 were read with the participant, which correspond to the care guided by the nurse.

### Statistical analysis

Initially, the data were described through measures such as mean, standard deviation, minimum, median and maximum (quantitative variables).

To analyze the relationship of the covariates of interest (age, relationship with the patient, gender, schooling, race and religion) and also to compare both groups and 3 moments of interest, a generalized linear model with Poisson distribution and identity link function was proposed. The class of generalized linear models is an extension of the traditional linear model that allows the population mean to be dependent on a linear predictor through a link function and allows for the probability distribution of the response variable to be any member of the exponential family (Normal Distribution, Binomial, Poisson and Gamma)^18^; the comparisons were performed using orthogonal contrasts. A Delta analysis was proposed to compare the difference between the EB and ST groups at t_2_ and t_0_. The Student’s t test was proposed in the comparison of the groups regarding the difference in the number of correct answers between t_0_ and t_2_ (Delta analysis).

The analyses was performed with the aid of the SAS 9.4^19^ software, adopting a 5% significance level.

### Ethical aspects

The paper was approved by the Research Ethics Committee of *Universidade Federal de São Carlos* - UFSCar, under CAAE No. 17428819.0.0000.5504 and opinion No. 3,556,901/2019, respecting the norms set forth in Resolution No. 466/12 of the National Health Council. All research participants signed two copies of the Free and Informed Consent Form (FICF), one being filed by one of the researchers (first author) and the other leaving in possession of the caregivers.

## Results

The final sample consisted of 30 (100%) caregivers that agreed to participate in all the stages; their age varied from 18 to 64 years old (M=44.4; SD=±14.4) and most of them (18 [60%]) were female. Of these, 20 (66.67%) were family members (son/daughter, wife, sibling, son/daughter-daughter-in-law) and 10 (33.33%) were not (caregivers and friends). In relation to schooling, it ranged between incomplete or lower Elementary School [14(46.67%)] and complete High School or Higher Education [16 (53.33%)]. 

In addition, the respondents identified themselves as white-skinned [11 (36.67%)] and as black- and/or brown-skinned [19 (63.33%)] and most of them stated being Catholics (60%).

Table 1 presents the description of the correct answers to the instrument prepared by the authors to evaluate the knowledge achieved by the population under study at the different research moments.

**Table 1:** Correct answers in the instrument to assess knowledge before and after the class and after applying the Educational Booklet (EB) and Skills Training (ST), (n=30). São Carlos, SP, Brazil, 2019 - 2020

Total of correct answers	n	Mean (SD)	Median (Q1 - Q3)	Min - Max
Before (t_0_)^*^	30	10.27 (4.91)	9 (7 - 13)	2 - 20
*EB*	15	10.67 (4.53)	10 (7 - 13)	3 - 20
*ST*	15	9.87 (5.38)	9 (6 - 13)	2 - 20
After the class (t_1_)^†^	30	17.87 (1.98)	18 (17 - 19)	13 - 20
*EB*	15	18.07 (1.67)	18 (17 - 19)	14 - 20
*ST*	15	17.67 (2.29)	18 (17 - 19)	13 - 20
After the interventions (t_2_)^‡^	30	19.47 (0.97)	20 (19 - 20)	16 - 20
*EB*	15	19.2 (1.26)	20 (19 - 20)	16 - 20
*ST*	15	19.73 (0.46)	20 (19 - 20)	19 - 20

*t_0_ = Before any intervention; ^†^t_1_ = After the LC intervention*;* ^‡^t_2_ = After the ST and EB interventions

**Figure 2:**
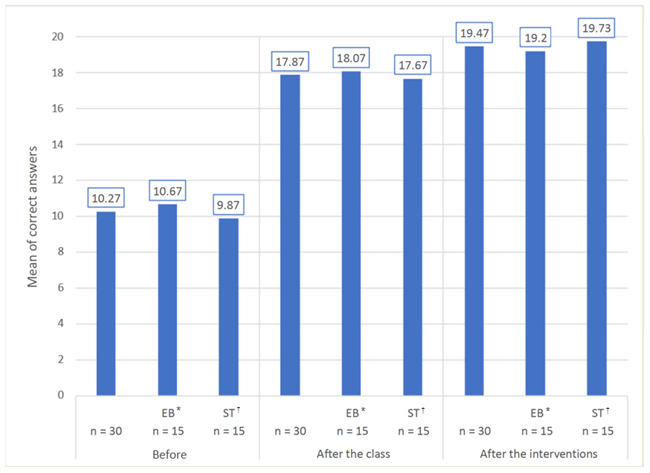
Histogram corresponding to the total of correct answers by period in which the instrument was applied. São Carlos, SP, Brazil, 2019 - 2020

^*^EB = Educational booklet; ^†^ST = Simulated skills training

Figure 2 was prepared in order to see the correct answers at the respective moments implemented in the research.

Figure 2 presents the mean of correct answers at the pre-intervention moment in relation to the total number of participants and by educational intervention, divided into EB and ST. It shows that the mean of correct answers given by the individuals presented a higher proportion after applying the first intervention. Given the above, the individuals showed a lower proportion of knowledge gain with the ST and the EB interventions, due to the previous knowledge acquired in class to carry out the interventions.

In the evaluation of the covariates, it was verified that there is evidence of a difference between the sex. The mean estimated difference was 2 correct answers, with the female gender (F) having more correct answers than the male gender (M). There was evidence of a difference across the races groups: white-skinned caregivers presented a mean of 1.60 more correct answers than their non-white-skinned counterparts.

It was identified that those with a family relationship (child, wife, sister, son-in-law, grandchild) with the patient had a mean gain of 1.19 more correct answers in the instrument when compared to those without family ties and friends of the patients. The other covariates, such as age, schooling and religion, did not present statistically significant differences.

Table 2 presents the comparison between the moments regardless of the interventions, and shows that there is a difference between the t_1_ and t_0_ moments. The estimated difference was 38 percentage points, with t_1_ presenting a higher percentage of correct answers.

**Table 2:** Comparison between the moments, regardless of the interventions. São Carlos, SP, Brazil, 2019 - 2020

Comparisons	Estimate	95% CI		p-value
t_1_^*^ - t_0_^†^	7.78	6.20	9.37	<0.01
t_2_^‡^ - t_0_	9.44	7.79	11.09	<0.01
t_2_ - t_1_	1.66	0.86	2.46	<0.01

*t_1_ = Moment after intervention LC (Lecture Class strategy); ^†^t_0_ = Moment before any intervention; ^‡^t_2_ = Moment after intervention ST (Skills Training) and intervention EB (Educational Booklet)

Table 3 compares the moments only considering the 15 caregivers who participated in the Educational Booklet, the 15 caregivers who participated in the Skills Training and the comparison between the groups at each specific time.

**Table 3:** Comparison of the estimate of correct answers between the moments considering the caregivers that participated in the EB interventions, those from the ST intervention, and the comparison between the groups at each specific moment. São Carlos, SP, Brazil, 2019 - 2020

Comparisons	Estimate	95% CI		p-value
EB group (t_1_^*^ - t_0_^†^)	7.44	5.24	9.63	<0.01
EB group (t_2_^‡^ - t_0_)	8.59	6.49	10.69	<0.01
EB group (t_2_ - t_1_)	1.15	0.07	2.23	0.04
ST group (t_1_ - t_0_)	8.13	5.84	10.42	<0.01
ST group (t_2_ - t_0_)	10.30	7.76	12.83	<0.01
ST group (t_2_ - t_1_)	2.17	0.97	3.36	<0.01
t_0_ (EB - ST)	0.60	-2.42	3.63	0.70
t_1_ (EB - ST)	-0.09	-1.66	1.47	0.91
t_2_ ST - t_1_	2.21	1.22	3.21	<0.01

*t_1_ = Moment after intervention LC (Lecture Class strategy); ^†^t_0_ = Moment before any intervention; ^‡^t_2_ = Moment after intervention ST (Skills Training) and intervention EB (Educational Booklet)

When comparing the moments regardless of the interventions, evidence of a difference between the t_1_ and t_0_ moments was noticed. The estimated difference was 38 percentage points, with t_1_ presenting a higher percentage of correct answers.

In turn, in the comparison between the moments and only considering the 15 caregivers that took part in EB and the 15 ones that participated in ST, it was evidenced that there was a difference between the t_2_ and t_1_ moments. The estimated difference is 1.15 correct answers, with the caregivers presenting a higher percentage of correct answers at t_2_. The results of Tables 2 and 3 were adjusted for age, relationship with the patient, sex, schooling, race and religion.

Table 4 presents the comparison of both groups regarding the difference in the number of correct answers between t_0_ and t_2_ performed by means of the Delta analysis.

**Table 4:** Descriptive analysis of the t_2_ - t_0_ difference between the Educational Booklet (EB) and Skills Training (ST) groups. São Carlos, SP, Brazil, 2019 - 2020

Difference between t_2_ and t_0_	n	Mean (SD)	Median (Q1 - Q3)	Min - Max
EB group (t_2_^*^ - t_0_^†^)	15	8.53 (4.42)	10 (4 - 11)	0 - 16
ST group (t_2_ - t_0_)	15	9.87 (5.29)	11 (6 - 14)	0 - 17

*t_2_ = Moment after the ST or EB interventions; ^†^t_0_ = Moment before any intervention

The analysis of the final comparison about the knowledge gain between the EB and ST groups, according to Student’s t, evidenced an estimated difference of -1,33 between EB and ST, with 95% CI (-4.98; 2.31) and p-value=0.46. Therefore, there was no evidence of a difference between the groups in relation to the t_2_ - t_0_ Delta. Thus, we cannot conclude that any of the groups changed more than the other in relation to the t_0_ and t_2_ moments.

Through a descriptive analysis of the instrument applied, it is observed through the simple mean of answers that after the LC that less than half of the participants [14 (46.6%)] were motivated, that 12 (40%) were self-confident and/or that 11 (36.6%) were confident. In this strategy (t_1_), 4 (13,3%) caregivers stated feeling “very little motivation”. In addition, 1 (6.6%) participant remained with “very little self-confidence” after the ST intervention and 1 (6.6%) was with “very little confidence” after the EB intervention.

After undergoing skills training, 9 (60%) of the participants classified themselves as “very confident”; 7 (46.6%) as “very self-confident” and 7 (33.3%) as “very motivated”. The caregivers who participated in the reading of the educational booklet had lower rates, with 4 (26.6%) considering themselves “very confident”, 1 (6.6%) caregiver “very self-confident” and 2 (13.3%) “very motivated”. In total (n=30), most of these participants classified themselves as motivated [11 (73.3%)], self-confident [9 (60%)] and secure [11 (73.3%)]. 

In relation to how all 30 participants felt after the LC intervention, 25 (83.3%) felt “good”, 5 (16,6%) stated being “tired’’ and none of them indicated feeling “bad”. After the ST intervention, 12 (80%) participants felt “good”, 2 (13.3%) felt “tired’’ and 1 (6.6%) felt “bad*”.* For the EB intervention participants, the results are similar to those of the aforementioned educational strategy: 12 (80%) felt “good”, 2 (13.3%) felt “tired’’ and 1 (6.6%) felt “bad”. For both strategies, an increase was observed in the self-perception of security, confidence and motivation for home-based care. Thirteen (43.3%) consider themselves “very motivated”, 8 (26.6%) “very self-confident” and 7 (23.3%) “very confident”, with no participant feeling “little motivated”.

## Discussion

The age of the caregivers participating in the study ranged from 18 to 64 years old, with a majority of females, and almost half with incomplete High School or lower schooling levels; similarly to the qualitative study that described the experiences of people living at home with Enteral Nutritional Therapy (ENT) in which caregivers were aged between 22 and 77 years old. For some authors^8^, aged caregivers with low schooling levels need more support and training from the multiprofessional team on enteral nutrition and care for patients with enteral tubes^20^.

Our findings corroborate promotion and preparation of the caregiver and all three interventions applied increased the participants’ knowledge, with a view to increasing the number of correct answers in the instrument at all stages. The studies^12^ ^,^ ^21^ reinforced the importance of guidance to caregivers carried out by professionals, as well as the development of health education methods that address the learning needs and particularities for adequate care and risk prevention for patients treated at their homes.

This process must be incorporated into hospital discharge planning and with an indication for home care, together with the participation of the multiprofessional team^1^ ^,^ ^22^. However, in the clinical practice, the authors notice that most of the information about HENT is provided at a specific moment and very close to hospital discharge. One study^14^ showed that the patients who are not part of a home-based follow-up enteral nutrition program face many challenges, including maintenance of functional status, complications inherent to enteral nutrition and lack of access to an interdisciplinary team, as well as the caregivers’ own competence.

The interactive lecture class can familiarize caregivers with the content and make it possible to instantly clarify doubts, in addition to stimulating dialogue and interaction between caregivers and family members with collective knowledge construction.

Studies that used simulation for NET practices, as discussed in 2020^2^, evidenced simulation as a very useful methodology to review good practices in NET in relation to skills training. This descriptive study^2^ used simulation to assess nursing technicians’ knowledge about potential complications and/or adverse events related to ENT in a simulated scenario and the clinical simulation allowed identifying risks in the practice of enteral nutrition therapy administration and means to minimize them.

Skills training as an enabler of teaching in different contexts confers skill and knowledge to the processes experienced during the learning phase, stimulating the use of clinical reasoning and planning of the care measures with the probe and its possible complications in a less abstract way^23^.

Using a scenario for skills training such as the one carried out in this study can help explore tube care and recognize possible complications in a less abstract way, emphasizing the importance of preventing errors and harms to the patients, helping to understand the process and reducing the level of anxiety associated with tube-related care, collaborating with the training of more active caregivers for safer home-based patient care^12^.

In addition to that, a number of authors^24^ emphasize that educational materials are capable of exploring resources that meet recognized and valued meanings in the context of users and the community, regardless of the cultural or social environment to which the individual belongs. It was observed that the educational booklet was a differential for the training of caregivers after the interactive lecture class, easing the approach to the content and ensuring that the important aspects were worked on, a fact that evidences the importance of investments of this nature.

One study^25^ indicated that individuals who receive educational materials attribute positive values to printed teaching materials. It also mention the relevance of the booklet as an important means of promoting knowledge in a clear format, with explanatory illustrations, which facilitate memorization of the necessary care measure, which may favor psychosocial aspects and positive behaviors.

Likewise, the educational manual assisted in the memorization of contents and in the ease of resuming what was discussed in the interactive lecture class, illustrating the tasks that can stimulate applicability of the material. Other authors^26^ emphasize that printed materials sensitize and educate the target audience, encompassing knowledge construction beyond the caregiver, collectively between the population and the professionals. For health professionals, this approach ensures accessibility and ease of use at all schooling levels, being a ready-to-use resource available at home for consultation in case of doubts^17^ ^,^ ^26^.

Our findings indicate that caregivers with family ties to the patient showed greater knowledge gains when compared to those without family ties. In a study^27^ it is shown that the home context can influence the care that is offered and, in the meantime, the family roles undergo changes to develop new care relationships with each other, added to the daily tasks that they already performed habitually^7^ ^,^ ^11^.

Regarding the different strategies, most of the participants stated not being motivated, confident, or secure to apply home-based care. Some authors^28^ explain that educational strategies for caregivers are useful tools in learning, enabling knowledge construction and reinforcing their skills. These resources should involve written information, face-to-face explanations, videos and demonstrations to help reduce anxiety and to improve the caregivers’ knowledge, attitudes and behavior for an adequate and possible response in situations involving caregiving in a home environment^23^. A study^29^ provided new evidence about the needs and experiences of caregivers of patients using HENT, highlighting the uncertainties about tube care, access to professionals during the transition and, mainly, about support to deal with routine and urgent problems with these patients.

Accordingly, a study^30^ that explored the experiences of caregivers and family members of patients using HENT identified that the participants felt unprepared, without sufficient training or education and without support from health professionals, without even knowing how to prepare EN or the correct position for feeding and feeding schedules, thus facing errors and resulting in negative consequences for the patient and the family.

The findings reveal that skills training created the opportunity for caregivers to better understand the care measures, knowing in the practice what was evident in our findings in relation to increased security, confidence and motivation of the participants in carrying out the care practices. As well as it corroborates the stimulus to autonomy and empowerment for decision-making, competence development, recognition of difficulties and problems that can arise from care through critical reflection for safe home-based care^31^.

This reflects that using different teaching strategies, and even these combined strategies, increases adequacy of the family members and caregivers, allowing them to complement each other and be more accurate and attractive. Thus, the guidance process, understanding and clarity are eased and there is increased accessibility to reach several age groups and schooling levels.

The nurse has a fundamental role in health education, in which they provide support for the transition to the home; however, the teaching and learning process also requires the participation of a multidisciplinary team for a more effective approach. It should be noted that caregivers are the main actors and must be active in this process, following the alignment of the ENT care plan to optimize, together with the team, the proposed results for the patient.

This study represents an important social contribution, with the active participation of caregivers, the education promoted by the multidisciplinary health team and, above all, the potential of nurse as care mediator. This is because the use of skills training in a simulated clinical scenario associated with verbal guidelines and with using educational manuals contributes considerable gains to the results of caregivers and/or family members, possibly by reducing abstraction of the guidelines^2^. However, it does have limitations, including the study sample and regionalization of results, as well as the use of non-validated evaluative questionnaires. In addition, there was no continuity in the evaluation of control of the complications or readmissions after the strategies applied.

## Conclusion

Knowledge was further increased between the t_1_ and t_0_ moments, when compared to the t_2_ and t_1_ moments in both groups. When compared, we cannot conclude that one of the groups changed more than the other in relation to moment t_0_ and t_2_; thus, the study evidenced the knowledge gain after all the educational strategies in both groups.

It is understood that using different educational strategies can collaborate with the training of more active, committed, stable and emotionally prepared caregivers in the resolution and analysis of problems, with critical decision-making of the practice and reduce the occurrence of complications resulting from use of this device; in addition to presenting significant contributions in the public policy context, facilitating access to information for users from different socioeconomic and cultural levels.

Health teams need to be encouraged to carry out planned hospital discharge; thus, it is considered that these findings can be useful for the educational development of patients and caregivers.
